# Dispensing of psychotropic medication among 400,000 immigrants in The Netherlands

**DOI:** 10.1007/s00127-017-1405-x

**Published:** 2017-06-14

**Authors:** Fabian Termorshuizen, Jean-Paul Selten, Eibert R. Heerdink

**Affiliations:** 1 Division of Pharmacoepidemiology and Clinical Pharmacology, Utrecht Institute for Pharmaceutical Sciences, Utrecht University, P.O. Box 80082, 3508 TB Utrecht, The Netherlands; 2 Rivierduinen, Institute for Mental Health Care, Sandifortdreef 19, 2333 ZZ Leiden, The Netherlands; 3 Department of Psychiatry and Psychology, School for Mental Health and Neuroscience MHeNS, Maastricht University, Universiteitssingel 40, 6229 ER Maastricht, The Netherlands; 4 Department of Clinical Pharmacy, University Medical Center Utrecht, Heidelberglaan 100, 3584 CX Utrecht, The Netherlands

**Keywords:** Ethnic minorities, Pharmacoepidemiology, Antipsychotics, Antidepressants, Psychostimulants

## Abstract

**Purpose:**

Previously, a high prevalence of certain psychiatric disorders was shown among non-Western immigrants. This study explores whether this results in more prescriptions for psychotropic medication.

**Methods:**

Data on dispensing of medication among adults living in the four largest Dutch cities in 2013 were linked to demographic data from Statistics Netherlands. Incident (i.e., following no dispensing in 2010–2012) and prevalent dispensing among immigrants was compared to that among native Dutch (*N* = 1,043,732) and analyzed using multivariable Poisson and logistic regression.

**Results:**

High adjusted Odds Ratios (OR_adj_) of prevalent and high Incidence Rate Ratios (IRR_adj_) of incident dispensing of antipsychotics were found among Moroccan (*N* = 115,455) and Turkish individuals (*N* = 105,460), especially among young Moroccan males (OR_adj_ = 3.22 [2.99–3.47]). Among Surinamese (*N* = 147,123) and Antillean individuals (*N* = 41,430), slightly higher rates of dispensed antipsychotics were found and the estimates decreased after adjustment. The estimates for antipsychotic dispensing among the Moroccan and Turkish increased, following adjustment for household composition. Rates for antidepressant dispensing among Turkish and Moroccan subjects were high (Moroccans: OR_adj_ = 1.74 [1.70–1.78]). Among Surinamese and Antillean subjects, the rates for antidepressant dispensing were low and the OR_adj_ lagged behind the IRR_adj_ (Surinamese: 0.69 [0.67–0.71] vs. 1.06 [1.00–1.13]). Similar results were found for anxiolytics. For ADHD medication, lower dispensing rates were found among all migrant groups.

**Conclusions:**

The findings agree with earlier reports of more mental health problems among Moroccan and Turkish individuals. Surinamese/Antillean individuals did not use psychotropic drugs at excess and discontinued antidepressants and anxiolytics earlier. The data strongly suggest under-treatment for ADHD in all ethnic minority groups.

## Introduction

The mental health of immigrants to the Western world is an important issue. The processes of loss, change, and finding ways to satisfy the new requirements of the host society may bring along various sources of stress and increased risks of mental health problems [1].

Different data sources can be used to quantify differences in the incidence and/or prevalence of mental health disorders between the native and migrant populations. Data from population surveys may be biased by the use of questionnaires that are not validated for certain ethnic minority groups, low response rates or high response rates from individuals with a higher level of education, and proficiency of the language of the host country [2]. Immigrants may underutilize mental health care, due to cultural and linguistic barriers, and mismatches between needs and provision of care [3]. As a consequence, the results from clinical records or other indicators of health care utilization, such as use of medication, may be influenced by these differences in help-seeking behavior and/or treatment compliance and, thus, may not yield valid figures. In a Spanish health region, contrary to the expectation of a higher risk of mental health problems, a lower use of antipsychotic and antidepressant medication was found among immigrants from Eastern Europe and sub-Saharan Africa [4, 5]. This may be explained by services not being adapted to the needs of immigrants, physicians’ attitudes, linguistic barriers, and/or cultural differences such as illness attributions and feelings of stigmatized [5, 6]. On the other hand, it is also possible that the prevalence of (mental) health problems among recent immigrants, such as those in this Spanish region, is low: the so-called ‘healthy immigrant effect’ [7, 8]. The prevalence of mental health problems may increase with the length of stay in the host country due to adverse factors such as socioeconomic hardship, discrimination, and disappointment with the perspectives on the labor market [9–13].

In The Netherlands, large and stable immigrant populations from four non-Western countries (Turkey, Morocco, Surinam, and Netherlands Antilles) have been present since a number of decades. An increased risk of mental disorders, especially schizophrenia among first- and second-generation individuals of Moroccan or Surinamese origin [14–16] and depressive disorder among subjects of Turkish origin [17–20], has been reported consistently. An analysis of data on dispensed psychotropic medication may be helpful to find out whether prescription rates are in accordance with the observed morbidity patterns and to monitor possible under-treatment [9].

An earlier Dutch study of data from a health-insurance company reported an increased rate of dispensed antipsychotic and antidepressant drugs among Moroccan and Turkish subjects and a lower rate of dispensed medication for attention deficit and hyperactivity disorder (ADHD) [21]. Unfortunately, those of Surinamese or Dutch Antillean origin were not considered. Furthermore, no distinction was made between incident and prevalent dispensing of medication. This may be important to reveal earlier cessation of use and lower treatment compliance among certain ethnic minorities, as was reported for the use of psychostimulants by Moroccan and Turkish children diagnosed with ADHD [22]. The present study used a large population-based data set to compare the incidence and prevalence of dispensing psychotropic medication to members of the four largest ethnic minority groups to that to Dutch natives.

## Methods

### Data sources

Two data sources were used. First, the population register (Dutch: Gemeentelijke Basis Administratie, GBA) present at Statistics Netherlands (Dutch: Centraal Bureau voor de Statistiek, CBS) records information on date of birth, sex, country of birth, country of birth of both parents, city and neighborhood of residence, marital status, and date of death for all legally residing citizens of The Netherlands. When a subject marries or changes his place of abode, a new record is added, but the historical records remain available for analysis.

The second database, from the Health Care Institute Netherlands (Dutch: Zorginstituut Nederland, ZiN), contains information on prescribed and dispensed medication reimbursed by health-insurance companies during the period 2006–2013. The data have been derived from all health-insurance companies in The Netherlands and have been collected for the purpose of risk adjustments, because some companies insure more patients with a high risk of expensive health care utilization than others. Dutch citizens are obliged by law to have a medical insurance. Since there is no distinction between public and private health-insurance companies, the results of our study are not influenced by selection of immigrants who can afford a health insurance. This database records information on drugs dispensed to outpatients and patients in nursing homes, not on drugs dispensed during episodes of in-patient treatment. For a particular calendar year and for a given individual, the first four positions of the ATC code of the medication(s) are registered. Dispensed medications within a calendar year with identical first four positions are mentioned only once for that subject. Thus, it is possible to establish whether a person had medication dispensed within a certain calendar year and for which class(es) of drugs (e.g., N06A, antidepressants; A10A: insulins and analogs).

Staff of CBS linked the information from the two databases, using the civil identification number, unique for each Dutch citizen. Dutch privacy laws allow the use of personal (health care) data for medical-scientific research without informed consent, provided that the results of the analysis cannot be traced to a unique person [23]. Consequently, staff of the CBS removed the postal code and the civil identification number from the files that were available to the researchers.

### Data extraction and definition of ethnic origin

To prevent the presence of correlated data because of more than one assessment of dispensing of a certain category of drugs within the same individual during subsequent years, the present analysis was restricted to one calendar year (2013). The analysis was also restricted to those aged 18 years and older and to persons resident during (a part of) this year in one of the four large cities in The Netherlands (Amsterdam, Rotterdam, The Hague, Utrecht). Most members of ethnic minorities in The Netherlands live in these cities, and this restriction made adjustments for population density or for urban–rural differences superfluous. Data on dispensed psychotropic medication (ATC codes N05, N06, N07B) were analyzed, leaving the use of medication for somatic disorders out of consideration. Characteristics of the household (i.e., being married, part of one-parent family, being single or living in an institute), as established at January 1st 2013, were regarded as time-independent during 2013. Socioeconomic status (SES) was established at the neighborhood level and defined as the proportion of households receiving national assistance benefit due to unemployment. If a person moved to another neighborhood in the same or another city during 2013, an average of this proportion for that person was estimated. This average was weighted for the duration of the subsequent episodes of residence during 2013 in the pertinent neighborhoods.

The CBS classifies ethnicity according to a citizen’s country of birth and the country of his or her parents’ birth. A Dutch-born subject with two Dutch-born parents is considered native Dutch. If a citizen has been born abroad, he or she is assigned to the ethnicity of the people born in the same country. A Dutch-born citizen is considered a second-generation citizen if at least one of his or her parents was foreign-born. If the parents were born in different foreign countries, the maternal country of birth was decisive for assignment to a particular ethnic group. Since most non-Western ethnic minorities in The Netherlands originate from Turkey, Morocco, Surinam, and the Netherlands Antilles, the analysis was restricted to these ethnic groups. Both first and second-generation immigrants were included according to the definition of ethnic minority group used by the CBS.

Labor migration from Morocco started in the 1960s. Many ‘guest workers’ decided to stay in The Netherlands and brought their families with them. Their SES is in general unfavorable compared to the Dutch majority, and they experience high degrees of discrimination. Turkish immigrants arrived in the same period, but report lower degrees of discrimination; they appear to have stronger bonds within their own ethnic group [24]. Surinam is a former Dutch colony that gained independence in 1975, which caused mass emigration to The Netherlands during the years thereafter. Most immigrants from the Netherlands Antilles, another former Dutch colony, moved to The Netherlands for study and work. The Surinamese population is racially diverse, while the Dutch Antillean population is predominantly black. Both populations speak Dutch fluently [25].

### Analysis

The prevalent dispensing of psychotropic medication in 2013 was analyzed as a yes/no variable in multivariable logistic regression models. The dispensing to members of any of the four ethnic minority groups was compared to the dispensing to the native Dutch in four separate analyses. The difference in proportions was expressed as an odds ratio (OR) with a 95% confidence interval (95% CI). The OR was adjusted for age and gender, and in a next step also for neighborhood SES and in a final step also for household composition. We distinguished between antipsychotics (N05A), antidepressants (N06A), anxiolytics (N05B), and psychostimulants (N06B).

The incident dispensing of psychotropic medication was analyzed using multivariable Poisson regression models. Incident dispensing in 2013 was defined as supply of a medication among subjects who had not filled a prescription for this particular category of psychotropic medication during the period 2010–2012. The difference in incidence rates (new users/1000 person-years) was expressed as an Incidence Rate Ratio (IRR), adjusted for age and gender, neighborhood SES, and household composition. The 95% CIs were adjusted for possible over-dispersion.

Data management and record linkage were performed with SPSS version 21.0. The logistic and Poisson regression analyses were performed with STATA, version 14.0.

## Results

### Description and crude figures on dispensing of psychotropic medication

Table 1 shows the numbers and characteristics of the included subjects, by ethnic group. There were major differences in age distribution, and SES of the neighborhood and household composition. Comparatively high numbers of Turkish–Dutch and Moroccan–Dutch subjects were married or co-habiting (70.2 and 67.0% vs 55.7%, *P* values <0.001), whereas high proportions of Surinamese and Antillean–Dutch subjects were members of a single-parent family (22.8 and 20.6% vs 5.8%, *P* values <0.001), which necessitate adjustments in the analyses.

**Table 1:** Profile of cohort of citizens in four largest Dutch cities by ethnic group: sociodemographic data and incident and prevalent dispensing of psychotropic drugs, 2013

*N* total	Dutch native	Turkish	Moroccan	Surinamese	Neth. Antillean
	*N* = 1,043,732	*N* = 105,460	*N* = 115,455	*N* = 147,123	*N* = 41,430
Age (years)					
18–30	250,888 (24.0%)	32,931 (31.2%)	36,855 (31.9%)	38,336 (26.0%)	16,654 (40.2%)
30–40	178,711 (17.1%)	27,625 (26.1%)	29,548 (25.5%)	27,571 (18.7%)	8691 (20.9%)
40–60	328,407 (31.4%)	34,763 (32.9%)	36,141 (31.3%)	58,532 (39.7%)	12,067 (29.1%)
≥60	285,726 (27.3%)	10,141 (9.6%)	12,911 (11.1%)	22,684 (15.4%)	4018 (9.7%)
Gender %male	507,138 (48.5%)	54,893 (52.0%)	59,857 (51.8%)	68,340 (46.4%)	20,297 (48.9%)
Unemployment rate neighborhood (SES)					
(#/1000 households) 1. <25	193,130 (18.5%)	2,646 (2.5%)	2816 (2.4%)	6107 (4.1%)	2528 (6.1%)
2. 25–51	231,580 (22.1%)	9385 (8.9%)	8373 (7.2%)	15,704 (10.6%)	4009 (9.6%)
3. 51–88	256,707 (24.6%)	11,997 (11.3%)	15,404 (13.3%)	25,212 (17.1%)	6978 (16.8%)
4.99–138	209,508 (20.0%)	27,740 (26.3%)	34,858 (30.1%)	35,022 (23.8%)	10,995 (26.5%)
5. ≥138	147,806 (14.1%)	53,451 (50.6%)	53,609 (46.4%)	64,560 (43.8%)	16,646 (40.1%)
9. unknown	5001 (0.4%)	241 (0.2%)	395 (0.3%)	518 (0.3%)	274 (0.6%)
Household characteristics					
Married/co-habiting	581,894 (55.7%)	74,081 (70.2%)	77,436 (67.0%)	63,287 (43.0%)	14,101 (34.0%)
Single-parent family	61,222 (5.8%)	10,808 (10.2%)	11,407 (9.8%)	33,679 (22.8%)	8570 (20.6%)
Unmarried/single	360,674 (34.5%)	16,183 (15.3%)	18,726 (16.2%)	42,741 (29.0%)	15,150 (36.5%)
Institute	20,619 (1.9%)	392 (0.3%)	979 (0.8%)	2429 (1.6%)	775 (1.8%)
9. Other/unknown	19,323 (1.8%)	3996 (3.7%)	6907 (5.9%)	4987 (3.3%)	2834 (6.8%)
Prevalence (%); incidence (*N*/1000py)					
All psychotropic drugs	11.36%; 27.56	14.51%; 42.04	14.19%; 40.14	11.10%; 32.77	7.43%; 27.67
Antipsychotics (N05A)	2.10%; 5.94	3.58%; 8.70	4.50%; 9.20	2.88%; 5.59	2.29%; 4.79
Antidepressants (N06A)	6.99%; 15.20	10.50%; 28.42	10.16%; 29.15	5.48%; 18.11	3.5%; 15.21
Anxiolytics (N05B)	2.95%; 10.06	4.50%; 20.26	3.73%; 15.18	3.86%; 15.08	2.05%; 10.38
Psychostimulants (N06B)	0.94%; 2.62	0.31%; 1.37	0.25%; 0.95	0.25%; 0.91	0.48%; 1.76

*py* person-years

Table 1 also shows crude figures on dispensed antipsychotics, antidepressants, anxiolytics, and psychostimulants for the separate ethnic groups.

### All psychotropic drugs (N05/N06/N07B)

#### Prevalence

The age- and gender-adjusted OR of filling a prescription for psychotropic medication (all classes) among the four ethnic minorities, compared to the native Dutch, mirrored the crude figures of Table 1 (Table 2). The results showed a significantly higher rate among the Turkish- (OR = 1.59) and Moroccan–Dutch (OR = 1.53), a similar rate among the Surinamese–Dutch (OR = 1.01), and a significantly lower rate among the Antillean–Dutch (OR = 0.76). After further adjustment for neighborhood SES, these ORs decreased significantly, as evidenced by 95% CIs that did not overlap the previous 95% CIs. Adjustment for household composition (in addition to age, gender, and SES) resulted in a further lowering of the OR among the Surinamese–Dutch and the Antillean–Dutch. Among the Turkish–Dutch and Moroccan–Dutch, on the other hand, the same adjustment resulted in a significant increase of the OR (to 1.54 and 1.50, respectively). Thus, this variable appears to be a protective factor for receiving a prescription for a psychotropic drug among Turkish–Dutch and Moroccan–Dutch, but a risk factor for the other two minorities, when comparing the model with adjustment for household composition and SES with the model with adjustment for SES (in addition to age and gender in all models). Indeed, being married or co-habiting (often found among the Turkish–Dutch and Moroccan–Dutch, see Table 1) was associated with a lower prevalence of psychotropic medication, whereas being part of a single-parent family (more frequent among the Surinamese–Dutch and the Antillean–Dutch) was associated with a higher prevalence of psychotropic medication (data available on request).

**Table 2:** Association between ethnic origin (ethnic minority group vs. Dutch natives) and prevalent/incident dispensing of psychotropic medication in four largest Dutch cities, 2013

	Prevalence			Incidence		
	OR [95% CI]	OR [95% CI]	OR [95% CI]	IRR [95% CI]	IRR [95% CI]	IRR [95% CI]
Adjustment for	Age and gender	Age and gender and SES^a^	Age, gender, SES, and household	Age and gender	Age and gender and SES^a^	Age, gender, SES, and household
All psychotropic drug (N05/N06/N07B)						
Turkish	1.59 [1.56–1.62]	1.38 [1.35–1.41]	1.54 [1.51–1.57]	1.79 [1.66–1.94]	1.58 [1.47–1.70]	1.66 [1.57–1.75]
Moroccan	1.53 [1.51–1.56]	1.34 [1.32–1.37]	1.50 [1.47–1.52]	1.66 [1.54–1.80]	1.48 [1.37–1.59]	1.55 [1.46–1.63]
Surinamese	1.01 [1.00–1.03]	0.90 [0.88–0.92]	0.88 [0.86–0.89]	1.27 [1.18–1.36]	1.14 [1.07–1.22]	1.10 [1.05–1.16]
Netherlands Antillean	0.76 [0.73–0.79]	0.68 [0.66–0.71]	0.64 [0.61–0.66]	1.16 [1.01–1.33]	1.05 [0.93–1.19]	0.98 [0.90–1.07]
Antipsychotic (N05A)						
Turkish	2.01 [1.94–2.08]	1.63 [1.57–1.69]	2.16 [2.08–2.25]	1.99 [1.65–2.39]	1.73 [1.43–2.10]	2.07 [1.85–2.32]
Moroccan	2.52 [2.44–2.60]	2.08 [2.01–2.15]	2.69 [2.60–2.79]	2.00 [1.68–2.40]	1.78 [1.48–2.14]	2.11 [1.89–2.36]
Surinamese	1.44 [1.40–1.49]	1.22 [1.18–1.27]	1.21 [1.17–1.25]	1.12 [0.93–1.35]	1.01 [0.84–1.22]	0.99 [0.89–1.11]
Netherlands Antillean	1.36 [1.27–1.45]	1.14 [1.07–1.22]	1.02 [0.95–1.09]	1.11 [0.77–1.60]	1.00 [0.69–1.43]	0.93 [0.76–1.14]
Antidepressant (N06A)						
Turkish	1.82 [1.78–1.86]	1.66 [1.62–1.70]	1.80 [1.76–1.84]	2.07 [1.93–2.22]	1.89 [1.76–2.03]	1.96 [1.84–2.09]
Moroccan	1.75 [1.72–1.79]	1.61 [1.57–1.64]	1.74 [1.70–1.78]	2.08 [1.94–2.23]	1.92 [1.79–2.06]	1.99 [1.87–2.11]
Surinamese	0.77 [0.75–0.79]	0.71 [0.69–0.73]	0.69 [0.67–0.71]	1.20 [1.12–1.29]	1.11 [1.04–1.18]	1.06 [1.00–1.13]
Netherlands Antillean	0.58 [0.55–0.61]	0.54 [0.51–0.57]	0.51 [0.48–0.54]	1.08 [0.95–1.24]	1.01 [0.89–1.14]	0.94 [0.84–1.06]
Anxiolytic (N05B)						
Turkish	1.93 [1.87–2.00]	1.53 [1.48–1.58]	1.76 [1.70–1.82]	2.35 [2.13–2.59]	1.94 [1.77–2.12]	2.06 [1.91–2.22]
Moroccan	1.58 [1.53–1.64]	1.27 [1.23–1.32]	1.46 [1.41–1.51]	1.74 [1.57–1.93]	1.46 [1.33–1.61]	1.54 [1.43–1.66]
Surinamese	1.41 [1.37–1.45]	1.16 [1.13–1.20]	1.13 [1.10–1.17]	1.58 [1.44–1.73]	1.36 [1.25–1.47]	1.32 [1.23–1.41]
Netherlands Antillean	0.91 [0.84–0.97]	0.75 [0.70–0.81]	0.69 [0.64–0.74]	1.22 [1.01–1.47]	1.05 [0.89–1.24]	0.98 [0.85–1.12]
Psychostimulant (N06B)						
Turkish	0.25 [0.23–0.28]	0.26 [0.23–0.29]	0.28 [0.25–0.31]	0.42 [0.35–0.51]	0.42 [0.35–0.52]	0.45 [0.38–0.54]
Moroccan	0.21 [0.19–0.24]	0.21 [0.19–0.24]	0.23 [0.20–0.26]	0.29 [0.24–0.36]	0.30 [0.24–0.37]	0.31 [0.26–0.38]
Surinamese	0.24 [0.21–0.26]	0.24 [0.22–0.27]	0.23 [0.21–0.25]	0.32 [0.26–0.38]	0.32 [0.27–0.39]	0.30 [0.25–0.36]
Netherlands Antillean	0.37 [0.32–0.43]	0.38 [0.33–0.44]	0.35 [0.30–0.41]	0.52 [0.40–0.67]	0.53 [0.41–0.69]	0.49 [0.39–0.62]

^a^Socioeconomic status (SES) defined as proportion of households in a neighborhood receiving national assistance benefit due to unemployment

#### Incidence

With incident dispensing as outcome, a similar picture was found for the Turkish–Dutch and Moroccan–Dutch. For the Surinamese–Dutch and the Antillean–Dutch, in contrast, IRRs significantly higher than 1.00 were obtained, after adjustment for age and gender. Again, these IRRs decreased following adjustment for consecutively neighborhood SES and household composition, but the 95% CIs remained above those of the matching ORs for the prevalence.

### Antipsychotics (N05A)

#### Prevalence

The ORs of receiving a prescription for antipsychotic medication among the Turkish–Dutch and Moroccan–Dutch, compared to the native Dutch, were all significantly higher than 1.00. Neighborhood SES explained only a part of the higher dispensing, and the ORs increased following adjustment for household composition. Among the Surinamese–Dutch and Antillean–Dutch, the prevalence was significantly higher than that among the Dutch natives, but the differences decreased considerably by adjusting for SES and household composition. Among the Surinamese–Dutch, the difference was still present and statistically significant after the adjustments (OR = 1.21 [1.17–1.25]).

#### Incidence

For the Turkish–Dutch and Moroccan–Dutch, a similar picture was obtained with incidence as outcome. Among the Surinamese–Dutch and the Antillean–Dutch, the IRRs did not significantly differ from 1, that is, no differences with the native Dutch were found.

### Antidepressants (N06A)

#### Prevalence

Among the Turkish–Dutch and the Moroccan–Dutch, the prevalence of antidepressants was significantly higher than that among the native Dutch. SES explained only a minor part of this difference. Again, adjustment for household composition suggested the presence of a protective effect of being married or co-habitation. Among the Surinamese–Dutch and Antillean–Dutch, the prevalence was significantly lower than that among the native Dutch, irrespective of adjustments (all ORs <1.00).

#### Incidence

For the Turkish–Dutch and the Moroccan–Dutch, the IRRs were somewhat higher than the ORs with non-overlapping 95% CIs. Remarkably, among the Surinamese–Dutch and Antillean–Dutch, the IRRs indicated no difference with the native Dutch, and the 95% CIs of the IRRs were all above the 95% CIs of the matching ORs. This suggests a shorter duration of treatment after starting antidepressants.

### Anxiolytics (N05B)

The incidence and prevalence rates of prescriptions for anxiolytics were higher among the Turkish–Dutch and the Moroccan–Dutch compared to the native Dutch, with similar effects of the various adjustments. Again, the IRRs were somewhat higher than the ORs with non-overlapping 95% CIs. Albeit less pronounced, an increased prevalence (OR = 1.13) and incidence (IRR = 1.32) were also obtained for the Surinamese–Dutch, after adjustments. In addition, the IRR was somewhat higher than the OR with non-overlapping 95% CIs. Among the Antillean–Dutch; however, a significantly lower prevalence (OR = 0.69) and a similar incidence (IRR = 0.98) were observed.

### Psychostimulants (N06B)

Among all ethnic minority groups, and irrespective of adjustments, the dispensing of psychostimulants was markedly lower. The IRRs were somewhat higher than the matching ORs, with no or minor overlap in 95% CIs, suggesting shorter treatment duration after starting psychostimulants among ethnic minority groups compared to the native Dutch.

### Subgroup analyses by gender and age (Tables 3, 4, 5, 6 in Appendix)

**Fig. 1:**
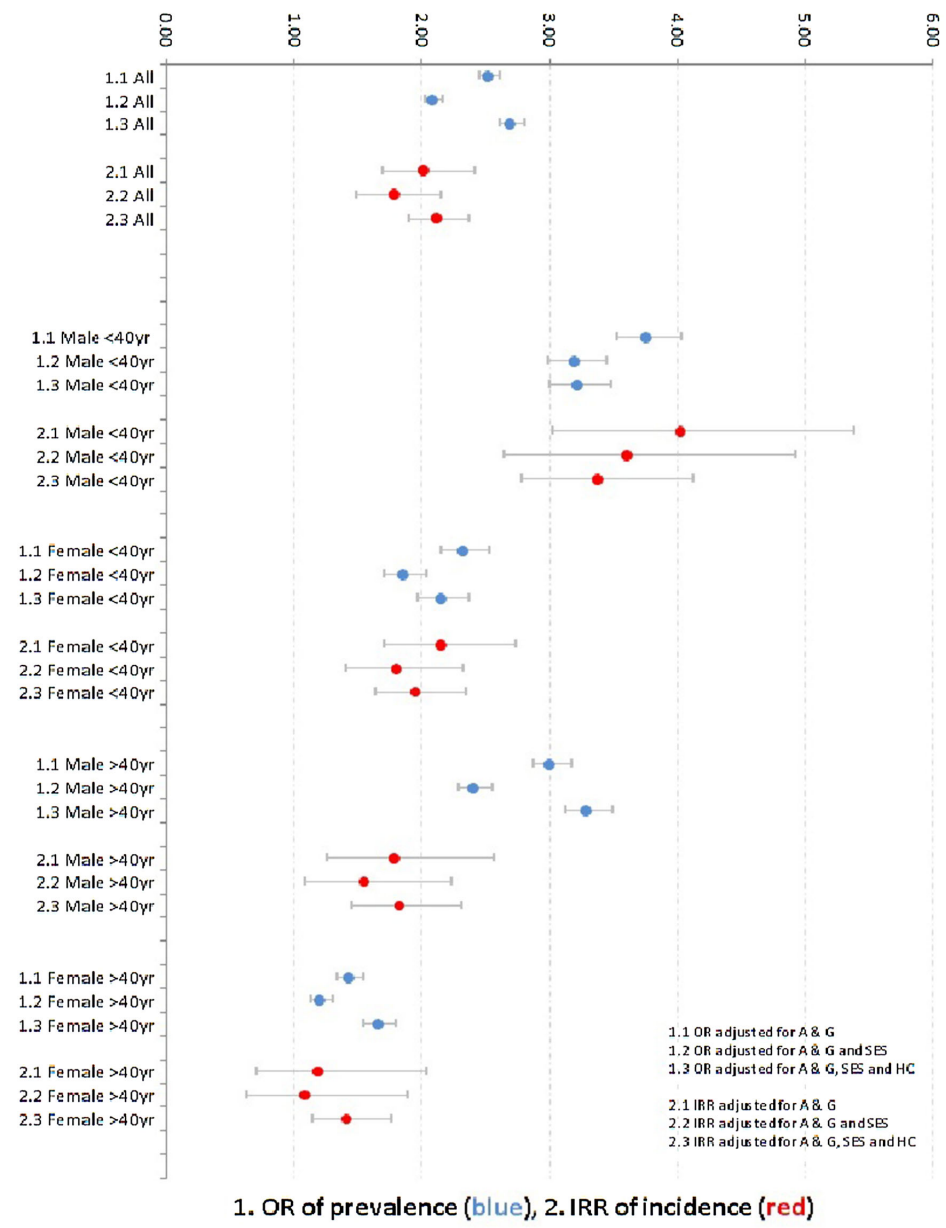
Odds ratios (ORs) and incidence rate ratios (IRRs) for dispensing of antipsychotic medication among all Moroccan–Dutch persons and for Moroccan–Dutch subgroups, defined by age and gender (vs. native Dutch); shown are 1.*x* ORs and 2.*x* IRRs, adjusted for *x*.1 age and gender (A&G), *x*.2 A&G and neighborhood socioeconomic status (SES), and *x*.3 A&G, SES, and household composition (HC)

Very high rates of dispensed antipsychotic medication were found among Moroccan–Dutch males aged <40 years (Fig. 1). Their adjusted OR (compared to native Dutch males aged <40 years) was 3.22 [2.99–3.47], and the adjusted IRR was 3.38 [2.77–4.11]. Among Moroccan–Dutch males aged ≥40 years, a high adjusted OR was found as well (3.29 [3.12–3.48]), whereas the IRR was considerably lower (1.82 [1.44–2.31]). The values related to antipsychotic medication for Moroccan–Dutch women of both age categories (compared to native Dutch females in the same age category) were substantially lower than those for Moroccan–Dutch males, but, after adjustment, still significantly higher than 1.0.

**Table 3:** Males <40 years (Dutch: *N* = 209,854; Turkish: *N* = 31,029; Moroccan: *N* = 32,340; Surinamese: *N* = 31,784; Antillean: *N* = 12,459)

	Prevalence			Incidence		
	OR [95% CI]	OR [95% CI]	OR [95% CI]	IRR [95% CI]	IRR [95% CI]	IRR [95% CI]
Adjustment for	Age and gender	Age and gender and SES	Age, gender, SES, and household	Age and gender	Age and gender and SES	Age, gender, SES, and household
All psychotropic drug (N05/N06/N07B)						
Turkish	1.21 [1.16–1.27]	1.11 [1.05–1.16]	1.20 [1.14–1.26]	1.66 [1.43–1.92]	1.51 [1.30–1.75]	1.56 [1.40–1.74]
Moroccan	1.62 [1.55–1.69]	1.50 [1.43–1.57]	1.53 [1.46–1.60]	1.87 [1.62–2.15]	1.70 [1.48–1.96]	1.68 [1.51–1.87]
Surinamese	0.92 [0.88–0.97]	0.86 [0.81–0.91]	0.80 [0.75–0.84]	1.12 [0.95–1.32]	1.04 [0.88–1.22]	0.99 [0.97–1.12]
Netherlands Antillean	0.68 [0.62–0.75]	0.64 [0.58–0.70]	0.56 [0.51–0.62]	0.92 [0.70–1.22]	0.86 [0.66–1.11]	0.80 [0.66–0.97]
Antipsychotic (N05A)						
Turkish	2.12 [1.96–2.30]	1.73 [1.58–1.88]	2.07 [1.89–2.26]	2.37 [1.69–3.32]	2.03 [1.43–2.89]	2.22 [1.81–2.73]
Moroccan	3.76 [3.52–4.02]	3.20 [2.98–3.44]	3.22 [2.99–3.47]	4.02 [3.01–5.38]	3.60 [2.64–4.92]	3.38 [2.77–4.11]
Surinamese	2.13 [1.96–2.31]	1.79 [1.65–1.95]	1.47 [1.34–1.61]	1.38 [0.91–2.10]	1.23 [0.81–1.87]	1.04 [0.81–1.32]
Netherlands Antillean	1.66 [1.45–1.90]	1.39 [1.21–1.60]	1.04 [0.90–1.20]	1.42 [0.74–2.71]	1.25 [0.67–2.35]	0.97 [0.68–1.39]
Antidepressant (N06A)						
Turkish	1.38 [1.29–1.47]	1.28 [1.20–1.37]	1.39 [1.30–1.49]	1.80 [1.54–2.10]	1.70 [1.44–2.01]	1.78 [1.56–2.04]
Moroccan	1.81 [1.71–1.91]	1.69 [1.59–1.79]	1.77 [1.66–1.88]	2.23 [1.93–2.57]	2.09 [1.80–2.44]	2.11 [1.86–2.40]
Surinamese	0.67 [0.62–0.73]	0.64 [0.58–0.69]	0.61 [0.56–0.67]	1.05 [0.87–1.28]	0.99 [0.82–1.20]	0.94 [0.80–1.11]
Netherlands Antillean	0.46 [0.39–0.54]	0.43 [0.37–0.51]	0.40 [0.34–0.47]	0.81 [0.57–1.16]	0.77 [0.54–1.09]	0.71 [0.54–0.94]
Anxiolytic (N05B)						
Turkish	2.13 [1.95–2.32]	1.76 [1.60–1.93]	1.95 [1.77–2.14]	2.67 [2.15–3.33]	2.20 [1.76–2.76]	2.34 [2.02–2.70]
Moroccan	2.28 [2.10–2.47]	1.95 [1.78–2.13]	1.91 [1.74–2.09]	2.49 [2.01–3.09]	2.10 [1.69–2.62]	2.00 [1.72–2.33]
Surinamese	1.46 [1.32–1.62]	1.27 [1.14–1.40]	1.09 [0.97–1.21]	1.73 [1.33–2.24]	1.51 [1.16–1.96]	1.36 [1.13–1.63]
Netherlands Antillean	0.93 [0.77–1.12]	0.80 [0.66–0.97]	0.64 [0.53–0.78]	1.39 [0.91–2.12]	1.20 [0.81–1.79]	1.03 [0.80–1.31]
Psychostimulant (N06B)						
Turkish	0.17 [0.14–0.21]	0.18 [0.15–0.22]	0.19 [0.15–0.23]	0.28 [0.20–0.39]	0.30 [0.21–0.43]	0.31 [0.23–0.42]
Moroccan	0.18 [0.15–0.21]	0.19 [0.15–0.22]	0.19 [0.15–0.23]	0.24 [0.16–0.35]	0.25 [0.17–0.38]	0.25 [0.18–0.35]
Surinamese	0.23 [0.20–0.27]	0.24 [0.20–0.28]	0.23 [0.20–0.28]	0.28 [0.20–0.40]	0.30 [0.21–0.42]	0.29 [0.22–0.39]
Netherlands Antillean	0.34 [0.28–0.42]	0.35 [0.28–0.43]	0.33 [0.27–0.41]	0.50 [0.33–0.76]	0.52 [0.34–0.80]	0.50 [0.35–0.71]

**Table 4:** Females <40 years (Dutch: *N* = 219,745; Turkish: *N* = 29,527; Moroccan: *N* = 34,063; Surinamese: *N* = 34,123; Antillean: *N* = 12,886)

	Prevalence			Incidence		
	OR [95%–CI]	OR [95%–CI]	OR [95%–CI]	IRR [95%–CI]	IRR [95%–CI]	IRR [95%–CI]
Adjustment for	Age and gender	Age and gender and SES	Age, gender, SES, and household	Age and Gender	Age and gender and SES	Age, gender, SES, and household
All psychotropic drug (N05/N06/N07B)						
Turkish	1.49 [1.43–1.55]	1.31 [1.25–1.36]	1.37 [1.31–1.43]	1.85 [1.61–2.12]	1.62 [1.41–1.86]	1.62 [1.47–1.78]
Moroccan	1.48 [1.42–1.53]	1.31 [1.26–1.36]]	1.37 [1.32–1.43]	1.87 [1.64–2.13]	1.66 [1.45–1.90]	1.66 [1.51–1.82]
Surinamese	0.89 [0.85–0.93]	0.81 [0.77–0.85]	0.72 [0.69–0.76]	1.30 [1.13–1.50]	1.17 [1.02–1.35]	1.04 [0.93–1.16]
Netherlands Antillean	0.73 [0.68–0.79]	0.66 [0.61–0.72]	0.57 [0.53–0.62]	1.25 [1.00–1.57]	1.12 [0.91–1.39]	0.95 [0.82–1.11]
Antipsychotic (N05A)						
Turkish	1.95 [1.78–2.14]	1.54 [1.40–1.71]	1.81 [1.63–2.00]	2.07 [1.57–2.73]	1.69 [1.26–2.26]	1.83 [1.48–2.25]
Moroccan	2.32 [2.14–2.52]	1.85 [1.69–2.03]	2.15 [1.96–2.36]	2.15 [1.69–2.73]	1.80 [1.40–2.32]	1.95 [1.63–2.34]
Surinamese	1.43 [1.30–1.58]	1.20 [1.08–1.33]	1.09 [0.98–1.22]	1.31 [0.98–1.75]	1.13 [0.83–1.52]	0.99 [0.79–1.25]
Netherlands Antillean	1.15 [0.97–1.36]	0.93 [0.78–1.11]	0.78 [0.65–0.94]	1.54 [1.00–2.37]	1.29 [0.85–1.97]	1.08 [0.80–1.45]
Antidepressant (N06A)						
Turkish	1.58 [1.51–1.66]	1.42 [1.35–1.49]	1.47 [1.40–1.55]	1.87 [1.62–2.15]	1.67 [1.44–1.93]	1.65 [1.48–1.85]
Moroccan	1.66 [1.59–1.74]	1.51 [1.44–1.58]	1.57 [1.50–1.65]	2.13 [1.88–2.43]	1.93 [1.68–2.21]	1.91 [1.73–2.12]
Surinamese	0.73 [0.69–0.78]	0.67 [0.63–0.71]	0.60 [0.56–0.64]	1.11 [0.95–1.29]	1.00 [0.86–1.16]	0.87 [0.77–0.99]
Netherlands Antillean	0.63 [0.57–0.69]	0.57 [0.52–0.64]	0.50 [0.45–0.56]	1.09 [0.86–1.40]	0.98 [0.78–1.24]	0.83 [0.70–0.98]
Anxiolytic (N05B)						
Turkish	2.35 [2.19–2.53]	1.87 [1.73–2.03]	1.93 [1.78–2.10]	2.49 [2.11–2.95]	2.05 [1.74–2.42]	2.03 [1.80–2.29]
Moroccan	1.80 [1.67–1.94]	1.45 [1.33–1.57]	1.52 [1.39–1.65]	1.91 [1.60–2.28]	1.59 [1.33–1.89]	1.59 [1.39–1.83]
Surinamese	1.50 [1.38–1.63]	1.26 [1.15–1.37]	1.08 [0.99–1.19]	1.71 [1.43–2.04]	1.47 [1.24–1.75]	1.29 [1.12–1.50]
Netherlands Antillean	1.01 [0.87–1.18]	0.84 [0.72–0.98]	0.67 [0.57–0.79]	1.23 [0.89–1.71]	1.06 [0.79–1.44]	0.88 [0.70–1.12]
Psychostimulant (N06B)						
Turkish	0.21 [0.17–0.26]	0.21 [0.17–0.26]	0.22 [0.18–0.28]	0.31 [0.21–0.46]	0.30 [0.20–0.45]	0.31 [0.22–0.42]
Moroccan	0.24 [0.20–0.29]	0,24 [0.20–0.29]	0.26 [0.21–0.31]	0.38 [0.26–0.54]	0.37 [0.26–0.54]	0.38 [0.28–0.52]
Surinamese	0.26 [0.22–0.31]	0.26 [0.22–0.31]	0.24 [0.20–0.29]	0.34 [0.24–0.50]	0.34 [0.23–0.51]	0.29 [0.21–0.40]
Netherlands Antillean	0.35 [0.27–0.45]	0.35 [0.27–0.45]	0.31 [0.24–0.40]	0.49 [0.29–0.82]	0.49 [0.29–0.82]	0.41 [0.26–0.63]

**Table 5:** Males >40 years (Dutch: *N* = 297,284; Turkish: *N* = 23,864; Moroccan: *N* = 27,517; Surinamese: *N* = 36.556; Antillean: *N* = 7838)

	Prevalence			Incidence		
	OR [95%–CI]	OR [95%–CI]	OR [95%–CI]	IRR [95%–CI]	IRR [95%–CI]	IRR [95%–CI]
Adjustment for	Age and gender	Age and gender and SES	Age, gender, SES, and household	Age and gender	Age and gender and SES	Age, gender, SES, and household
All psychotropic drug (N05/N06/N07B)						
Turkish	1.80 [1.73–1.86]	1.54 [1.48–1.60]	1.81 [1.74–1.88]	1.77 [1.50–2.09]	1.57 [1.35–1.82]	1.66 [1.52–1.83]
Moroccan	2.00 [1.94–2.07]	1.72 [1.66–1.78]	2.01 [1.94–2.07]	1.55 [1.30–1.84]	1.38 [1.17–1.62]	1.47 [1.32–1.63]
Surinamese	1.26 [1.22–1.30]	1.11 [1.07–1.15]	1.07 [1.04–1.11]	1.34 [1.16–1.55]	1.21 [1.06–1.38]	1.19 [1.10–1.29]
Netherlands Antillean	0.95 [0.88–1.02]	0.84 [0.78–0.90]	0.74 [0.69–0.80]	1.23 [0.90–1.68]	1.11 [0.84–1.46]	1.06 [0.90–1.24]
Antipsychotic (N05A)						
Turkish	2.09 [1.97–2.22]	1.66 [1.56–1.77]	2.35 [2.20–2.51]	1.77 [1.21–2.60]	1.51 [1.03–2..22]	1.79 [1.41–2.29]
Moroccan	3.01 [2.86–3.16]	2.41 [2.28–2.54]	3.29 [3.12–3.48]	1.78 [1.25–2.55]	1.55 [1.08–2.23]	1.82 [1.44–2.31]
Surinamese	1.55 [1.46–1.64]	1.29 [1.21–1.36]	1.21 [1.14–1.28]	1.14 [0.79–1.66]	1.02 [0.70–1.48]	0.99 [0.80–1.24]
Netherlands Antillean	1.55 [1.39–1.74]	1.28 [1.14–1.43]	1.02 [0.90–1.14]	0.89 [0.37–2.15]	0.79 [0.34–1.86]	0.71 [0.43–1.17]
Antidepressant (N06A)						
Turkish	2.16 [2.07–2.25]	1.96 [1.88–2.05]	2.20 [2.11–2.30]	2.23 [1.94–2.57]	2.02 [1.76–2.31]	2.16 [1.94–2.40]
Moroccan	2.41 [2.32–2.50]	2.18 [2.10–2.27]	2.43 [2.33–2.52]	2.16 [1.87–2.49]	1.97 [1.71–2.26]	2.10 [1.87–2.35]
Surinamese	0.89 [0.85–0.93]	0.82 [0.78–0.86]	0.80 [0.77–0.84]	1.28 [1.12–1.46]	1.18 [1.04–1.34]	1.16 [1.05–1.29]
Netherlands Antillean	0.58 [0.51–0.65]	0.53 [0.47–0.60]	0.49 [0.44–0.56]	1.04 [0.77–1.42]	0.96 [0.73–1.27]	0.91 [0.74–1.13]
Anxiolytic (N05B)						
Turkish	1.88 [1.77–1.99]	1.49 [1.40–1.59]	1.83 [1.71–1.95]	2.05 [1.67–2.52]	1.74 [1.43–2.11]	1.92 [1.69–2.18]
Moroccan	1.86 [1.76–1.97]	1.49 [1.40–1.58]	1.79 [1.69–1.90]	1.61 [1.30–2.00]	1.38 [1.12–1.68]	1.50 [1.31–1.72]
Surinamese	1.52 [1.44–1.60]	1.26 [1.19–1.33]	1.21 [1.14–1.28]	1.67 [1.40–2.00]	1.45 [1.23–1.71]	1.41 [1.27–1.57]
Netherlands Antillean	0.96 [0.84–1.10]	0.79 [0.69–0.90]	0.68 [0.60–0.78]	1.18 [0.76–1.82]	1.02 [0.70–1.51]	0.94 [0.74–1.21]
Psychostimulant (N06B)						
Turkish	0.49 [0.39–0.61]	0.50 [0.40–0.62]	0.55 [0.44–0.70]	0.98 [0.73–1.31]	0.95 [0.70–1.28]	1.03 [0.78–1.36]
Moroccan	0.28 [0.21–0.37]	0.29 [0.22–0.38]	0.32 [0.24–0.42]	0.31 [0.20–0.48]	0.31 [0.20–0.49]	0.33 [0.22–0.50]
Surinamese	0.22 [0.17–0.29]	0.23 [0.17–0.30]	0.22 [0.17–0.29]	0.38 [0.26–0.56]	0.39 [0.27–0.57]	0.38 [0.27–0.54]
Netherlands Antillean	0.47 [0.32–0.69]	0.48 [0.32–0.70]	0.44 [0.30–0.65]	0.39 [0.19–0.83]	0.40 [0.19–0.83]	0.37 [0.19–0.73]

**Table 6:** Females >40 years (Dutch: *N* = 316,849; Turkish: *N* = 21,040; Moroccan: *N* = 21,535; Surinamese: *N* = 44,660; Antillean: *N* = 8247)

	Prevalence			Incidence		
	OR [95%–CI]	OR [95%–CI]	OR [95%–CI]	IRR [95%–CI]	IRR [95%–CI]	IRR [95%–CI]
Adjustment for	Age and gender	Age and gender and SES	Age, gender, SES, and household	Age and gender	Age and gender and SES	Age, gender, SES, and household
All psychotropic drug (N05/N06/N07B)						
Turkish	1.73 [1.68–1.79]	1.50 [1.45–1.55]	1.65 [1.60–1.71]	1.89 [1.57–2.27]	1.64 [1.39–1.95]	1.75 [1.57–1.97]
Moroccan	1.16 [1.12–1.20]	1.01 [0.97–1.05]	1.12 [1.08–1.16]	1.45 [1.20–1.75]	1.27 [1.07–1.51]	1.36 [1.22–1.51]
Surinamese	0.95 [0.93–0.98]	0.84 [0.82–0.86]	0.82 [0.80–0.84]	1.27 [1.11–1.46]	1.13 [1.00–1.28]	1.10 [1.02–1.19]
Netherlands Antillean	0.72 [0.67–0.77]	0.64 [0.60–0.68]	0.61 [0.57–0.65]	1.19 [0.88–1.61]	1.07 [0.83–1.39]	1.01 [0.86–1.19]
Antipsychotic (N05A)						
Turkish	1.90 [1.78–2.03]	1.58 [1.48–1.69]	2.13 [1.98–2.29]	1.97 [1.27–3.06]	1.78 [1.12–2.82]	2.28 [1.85–2.82]
Moroccan	1.43 [1.33–1.54]	1.21 [1.12–1.30]	1.66 [1.53–1.79]	1.19 [0.69–2.03]	1.08 [0.62–1.88]	1.41 [1.13–1.76]
Surinamese	1.18 [1.11–1.25]	1.02 [0.96–1.08]	1.04 [0.98–1.11]	0.99 [0.67–1.46]	0.91 [0.61–1.36]	0.92 [0.78–1.07]
Netherlands Antillean	1.12 [0.99–1.27]	0.97 [0.85–1.10]	0.96 [0.84–1.10]	0.87 [0.34–2.20]	0.80 [0.31–2.04]	0.79 [0.56–1.11]
Antidepressant (N06A)						
Turkish	1.95 [1.88–2.02]	1.79 [1.72–1.86]	1.90 [1.83–1.98]	2.37 [2.08–2.70]	2.18 [1.92–2.47]	2.25 [2.00–2.52]
Moroccan	1.33 [1.28–1.39]	1.23 [1.18–1.28]	1.31 [1.26–1.36]	1.88 [1.65–2.14]	1.75 [1.54–1.99]	1.80 [1.61–2.02]
Surinamese	0.75 [0.72–0.78]	0.69 [0.67–0.72]	0.68 [0.65–0.70]	1.27 [1.14–1.41]	1.18 [1.07–1.30]	1.13 [1.04–1.24]
Netherlands Antillean	0.60 [0.56–0.66]	0.56 [0.52–0.61]	0.54 [0.50–0.59]	1.27 [1.02–1.59]	1.20 [0.98–1.47]	1.14 [0.94–1.37]
Anxiolytic (N05B)						
Turkish	1.75 [1.66–1.85]	1.37 [1.29–1.45]	1.54 [1.46–1.63]	2.36 [1.94–2.86]	1.91 [1.62–2.25]	2.02 [1.76–2.32]
Moroccan	1.06 [0.99–1.13]	0.84 [0.79–0.90]	0.95 [0.89–1.01]	1.41 [1.14–1.75]	1.16 [0.98–1.38]	1.23 [1.07–1.41]
Surinamese	1.33 [1.28–1.39]	1.09 [1.04–1.14]	1.06 [1.02–1.11]	1.45 [1.24–1.69]	1.23 [1.08–1.40]	1.20 [1.08–1.34]
Netherlands Antillean	0.83 [0.74–0.93]	0.69 [0.61–0.77]	0.65 [0.58–0.72]	1.17 [0.82–1.67]	1.00 [0.75–1.34]	0.94 [0.75–1.19]
Psychostimulant (N06B)						
Turkish	0.55 [0.43–0.72]	0.57 [0.44–0.75]	0.60 [0.46–0.79]	0.79 [0.51–1.23]	0.80 [0.52–1.24]	0.84 [0.59–1.19]
Moroccan	0.20 [0.13–0.30]	0.20 [0.13–0.31]	0.22 [0.14–0.33]	0.27 [0.14–0.52]	0.28 [0.15–0.54]	0.30 [0.18–0.49]
Surinamese	0.25 [0.19–0.32]	0.26 [0.20–0.34]	0.23 [0.17–0.30]	0.30 [0.19–0.48]	0.31 [0.20–0.49]	0.28 [0.19–0.40]
Netherlands Antillean	0.58 [0.39–0.87]	0.60 [0.40–0.91]	0.50 [0.33–0.76]	0.93 [0.52–1.65]	0.98 [0.56–1.70]	0.82 [0.54–1.24]

Among the Turkish–, the Surinamese–, and the Antillean–Dutch, in contrast, no pronounced differences in adjusted ORs and IRRs across age and gender strata were found.

**Fig. 2:**
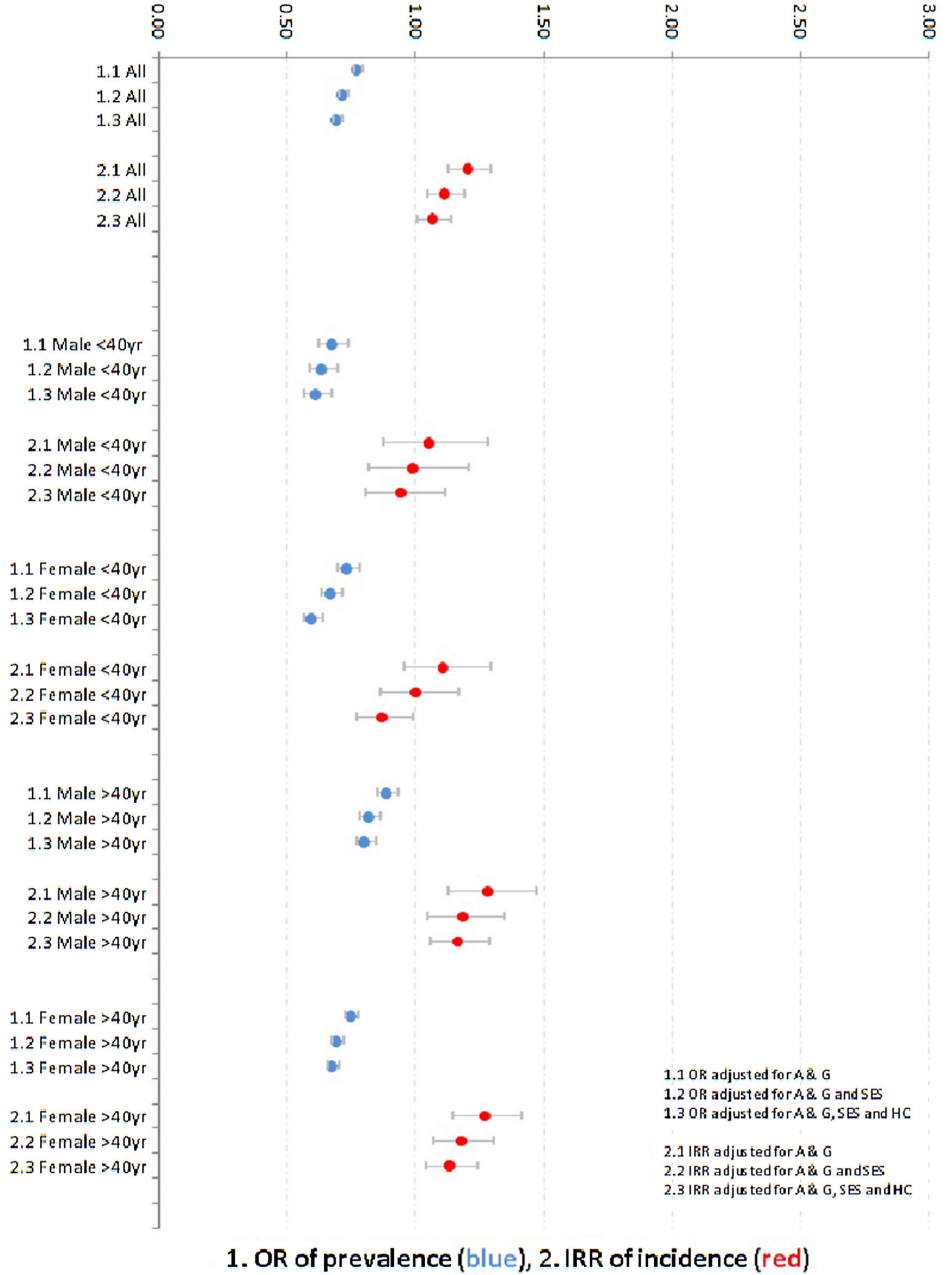
Odds ratios (ORs) and incidence rate ratios (IRRs) for dispensing of antidepressants among all Surinamese–Dutch persons and among Surinamese-Dutch subgroups, defined by age and gender (vs. native Dutch); shown are 1.*x* ORs and 2.*x* IRRs, adjusted for *x*.1 age and gender (A&G), *x*.2 A&G and neighborhood socioeconomic status (SES), and *x*.3 A&G, SES, and household composition (HC)

The discrepancies between the incidence and prevalence of antidepressants dispensing were found in most age and gender strata, especially among the Surinamese–Dutch (Fig. 2) and Antillean–Dutch. Comparatively high ORs and IRRs and—as an exception—a reverse pattern of a lower IRR than the matching OR were found for antidepressants among the Turkish–Dutch and Moroccan–Dutch males aged ≥40 years.

A high prevalence of dispensing of anxiolytic medication was found among Moroccan males aged <40 years (OR = 1.91 [1.74–2.09] and IRR = 2.00 [1.72–2.33]), whereas this prevalence among Moroccan females aged ≥40 years was comparatively low (OR = 0.95 [0.89–1.01] and IRR = 1.23 [1.07–1.41]). Among the Antillean–Dutch, similar or lower dispensing of anxiolytic medication was found across the four age–gender strata, with IRRs higher than the matching ORs.

The lower prevalence and incidence of prescriptions of psychostimulants among the ethnic minority groups compared to the native Dutch were found in almost all age–gender strata. As an exception, among Turkish–Dutch males aged ≥40 years, the IRR was 1.03 [0.78–1.36]. The matching OR, however, was significantly lower (0.55 [0.44–0.70]. In most groups, the IRR was higher than the matching OR.

## Discussion

This study found comparatively high dispensing rates for antipsychotics, antidepressants, and anxiolytics among the Moroccan–Dutch and Turkish–Dutch, while the corresponding rates among the Surinamese–Dutch and Antillean–Dutch were less pronounced. Second, IRRs of incident dispensing higher than the matching ORs of prevalent dispensing of antidepressants and anxiolytics, especially among the Surinamese–Dutch and the Antillean–Dutch were found. As the prevalence is a result of both the incidence and the duration of a certain condition (here treatment with antidepressants and anxiolytics), this finding points to earlier cessation of these drugs among the Surinamese–Dutch and the Antillean–Dutch compared to the native Dutch. This earlier cessation primarily suggests a worse compliance with the prescribed medication but may also be due to a more favorable course of the disorder. A third remarkable finding was the influence of the composition of the household. This turned out to be a favorable factor among the Turkish–Dutch and Moroccan–Dutch, but an unfavorable one among their Surinamese and Antillean counterparts. Those of Surinamese and Antillean origin appeared to be more often part of a one-parent family, and being part of an one-parent family was associated with the multivariable models with a higher dispensing rate of antipsychotics, antidepressants, anxiolytics, and psychostimulants compared to being part of a household with a married couple (data available on request). Finally, the dispensing rates for psychostimulants were considerably lower among all ethnic minority groups (ORs and IRRs <0.50).

### Comparison with previous studies

A recent meta-analysis showed that the psychosis risks for Moroccan–Dutch males are significantly higher than those for Moroccan–Dutch females [26]. Possible explanations for this hitherto unexplained phenomenon are the higher risk of illicit drug use among Moroccan–Dutch males and socioenvironmental risk factors such as achievement–expectation mismatch, discrimination, and stigmatization [26]. The increased dispensing rates of antipsychotics among the Moroccan–Dutch and the major gender gap herein as found in the present study are in accordance with the pertinent epidemiological studies conducted in The Netherlands [15, 16, 25–27]. We have no ready explanation for the much less increased OR of dispensed antipsychotic medication among the Moroccan–Dutch reported by Wittkampf et al. (OR = 1.15 [1.10–1.21]) [21].W Whether this is due to restriction of individuals at one certain insurance company remains a question.

As the mentioned studies focus on different links in the chain from the true prevalence/incidence of psychopathology, health seeking and diagnosis, hospital admission, medication prescription, to medication dispensing, actual use, and treatment compliance, comparison of these studies has to be done with caution and agreement does not necessarily preclude the presence of inconsistencies and, the other way round, lack of agreement does not necessarily indicate inconsistencies.

A remarkable finding in our study was the significantly increased dispensing of antipsychotic medication among the Turkish–Dutch. Epidemiological studies of non-affective psychotic disorder have reported results that differed by generation: a significant increase in risk for the second generation [16, 18, 28], not for the first [16, 25]. As antipsychotics are often prescribed for other indications, the increased use among the Turkish aged 40 years and older may be due to prescriptions in patients with dementia [29] and among the Turkish of all ages for treatment of depressive disorders. Indeed, there have been consistent reports in the literature of an increased risk of depressive disorders among the Turkish–Dutch [17–20, 30], and this may explain the higher dispensing rates of both antipsychotic, antidepressant, and anxiolytic medication in this ethnic group.

Higher risks for non-affective psychotic disorder have also been reported for Surinamese and Antillean immigrants to The Netherlands [14–16, 25, 27]. For example, a national register-based study reported an age- and gender-adjusted RR of the first admission for schizophrenia of 3.80 [3.52–4.10] for Surinamese immigrants, and of 3.98 [3.50–4.53] for Antillean immigrants [14]. More recently, a register-based study from the region of Utrecht found higher Relative Risks of non-affective psychotic disorders for the combined Surinamese and Antillean populations, which ranged from 2.12 to 3.44 [27]. These effect measures suggest that the dispensing of antipsychotic medication, as assessed in the present study, does not match the true occurrence of psychotic disorders. This may indicate under-treatment and/or worse compliance with psychotropic medication in general, as there was also a clear discrepancy between incident and prevalent use of antidepressants and anxiolytic drugs in these ethnic groups. A worse treatment compliance for antidepressants has been reported before for non-Western minorities in The Netherlands [31, 32]. Another interesting possibility, however, is a decreasing incidence of psychotic disorder among the Surinamese–Dutch and Antillean–Dutch over time. This interpretation is suggested by relatively higher prevalence rates for use of antipsychotics than incidence rates. Future studies should resolve this important issue.

Interestingly, the effects of adjustment for household composition on the risk for dispensing of antipsychotics among the Surinamese–Dutch and Antillean–Dutch support the report of a major effect of parental separation on the risk for psychosis among African-Caribbeans in the UK [33].

The results with regard to psychostimulants (almost always prescribed for ADHD), confirm the reports by Wittkampf et al. (2010) [21] and van der Ban et al. (2015) [22]. Lower use of ADHD medication by members of ethnic minorities has also been reported in USA [34, 35]. There may be a higher treatment threshold and/or a greater tolerance of behavioral problems among ethnic minorities [36, 37]. Our results show that the lower utilization of ADHD medication among ethnic minorities is not restricted to children but is also clearly present among adults of all age categories.

### Strengths and limitations

Our population-based study included a very large sample of more than one million native Dutch individuals and more than 400,000 first- and second-generation immigrants. It used administrative data of high quality that was fully independent of willingness or ability to participate in surveys and that was not influenced by any recall bias. The use of data from Statistics Netherlands guaranteed a correct identification of ethnic origin.

A number of limitations, however, have to be noted. The data were available in a crude format, indicating use of medication in broad ATC categories, but without details on dosage, prescribing specialism, and clinical diagnosis. Registered medication with ATC code N05A for antipsychotics also includes lithium (N05AN), which is a mood stabilizer, not an antipsychotic. Consequently, since evidence suggests that the Moroccan–Dutch and Turkish–Dutch use lithium less frequently than the native Dutch, the present figures may underestimate the ethnic gap in the use of antipsychotics [21]. Furthermore, antidepressants may be prescribed for anxiety disorders and antipsychotic drugs are also prescribed for non-psychotic disorders such as sleep disorders and behavioral problems associated with dementia and for psychotic mood disorders. Thus, the observation of a higher dispensing of antipsychotic medication by Moroccan–Dutch individuals does not necessarily point to an adequate treatment of those with a diagnosis of non-affective psychotic disorder or to a higher incidence/prevalence of psychotic disorders in this ethnic group. Still, it is in accordance with earlier studies that showed a higher frequency of mental health problems among the Moroccan–Dutch.

The design of our study was cross-sectional with a binary measure of medication dispensing in 2013. Defining episodes of medication use with starting and finishing dates and intermediate episodes of no use would make it possible to study ethnic differences in starting treatment and adherence in more detail. Still, the data on subsequent calendar years allowed of a comparison between crude measures for prevalent and incident use, which brought to light important differences in treatment adherence. The information from Statistics Netherlands enabled us to adjust for two important determinants of mental health care utilization: SES and household composition. However, SES was measured at the neighborhood level, not at the level of the individual. Nevertheless, our results agree with those of earlier studies, which showed that the relative risk of psychiatric disorder for members of ethnic minorities is reduced by adjustment for SES [38].

Household composition was handled as time-fixed, but this may be regarded as sufficient for the short observation period. The association with household composition is an interesting finding. This warrants further exploration, e.g., to find out whether the so-called ethnic density effect (i.e., a lower prevalence of certain mental health outcomes for members of ethnic minorities in neighborhoods with a higher concentration of members of their own ethnic group [27, 39] is (partly) mediated by this variable.

A limitation of the study was the relatively small number of co-variables available for adjustments. However, this study was not an in-depth etiological study aiming at establishing causal relationships between different ethnic origins and incidence of mental health problems, but to check whether results from population-based administrative databases on medication dispensing confirm epidemiological findings derived from smaller samples. The available variables (age, gender, SES, household composition and ethnicity) may be regarded as the most important to characterize individuals in studies of this kind. Of note, many epidemiological studies on ethnic differences in risk of mental health problems adjusted the results for age and gender only [9].

### Conclusion and relevance

The observed ethnic pattern in the dispensing of psychotropic medication at the population level is to a certain degree in accordance with the pattern reported by earlier studies. However, the findings also strongly suggest under-treatment of psychotic and depressive disorders among the Surinamese–Dutch and Antillean–Dutch and of ADHD among all ethnic minority groups. Thus, data on use of psychotropic medication at the population level are useful for addressing ethnic disparities in mental health care utilization and for the evaluation of public health interventions that aim to adjust these disparities.

## Appendix

Association between ethnic origin (ethnic minority group vs. Dutch natives) and prevalent/ incident dispensing of psychotropic medication in four largest Dutch cities, by age and gender strata, 2013 (Tables 3, 4, 5, 6).
